# Elicitation and aggregation of multimodal estimates improve wisdom of crowd effects on ordering tasks

**DOI:** 10.1038/s41598-024-52176-3

**Published:** 2024-02-01

**Authors:** Yeawon Yoo, Adolfo R. Escobedo, Ryan Kemmer, Erin Chiou

**Affiliations:** 1 The Martin V. Smith School of Business & Economics, California State University Channel Islands, 1 University Drive, Camarillo, CA 93012 USA; 2 Edward P. Fitts Department of Industrial and Systems Engineering, North Carolina State University, 915 Partners Way, Raleigh, NC 27606 USA; 3 School of Computing and Augmented Intelligence, Arizona State University, P.O. Box 878809, Tempe, AZ 85281 USA; 4 The Polytechnic School, Arizona State University, 7271 E Sonoran Arroyo Mall, Mesa, AZ 85212 USA

**Keywords:** Human behaviour, Mathematics and computing

## Abstract

We present a wisdom of crowds study where participants are asked to order a small set of images based on the number of dots they contain and then to guess the respective number of dots in each image. We test two input elicitation interfaces—one elicits the two modalities of estimates jointly and the other independently. We show that the latter interface yields higher quality estimates, even though the multimodal estimates tend to be more self-contradictory. The inputs are aggregated via optimization and voting-rule based methods to estimate the true ordering of a larger universal set of images. We demonstrate that the quality of collective estimates from the simpler yet more computationally-efficient voting methods is comparable to that achieved by the more complex optimization model. Lastly, we find that using multiple modalities of estimates from one group yields better collective estimates compared to mixing numerical estimates from one group with the ordinal estimates from a different group.

## Introduction

The wisdom of crowds (WOC) phenomenon, which posits that the aggregate information from a group can surpass the accuracy of any individual, inclusive of subject-matter experts, has been observed across various contexts. A compelling popular example comes from the television game show “Who wants to be a millionaire”, where contestants are offered opportunities to consult an expert and solicit the collective responses from a live studio audience to answer a given question. Over the show’s height in popularity, expert answers were reported to be correct 65% of the time, while audiences were correct 91% of the time^1^. Examples of the WOC phenomenon in other settings include identifying and classifying craters on the surface of Mars^2^, estimating corporate earnings^3^, predicting winners of elections^4^, estimating the height of a mountain^5^, among many others.

The vast majority of studies to assess crowd wisdom involve *numerical estimation* (i.e., numerosity) tasks such as guessing how many jelly beans are packed inside a glass jar^6^ and estimating the number of black dots on a white background^7^. However, prior works have also evaluated its applicability to *ordering* tasks, such as recalling the correct ordering of a list after it is shuffled^8,9^ and ordering multiple images based on the number of dots they contain (i.e., from the image with the fewest to the one with the most)^10,11^. But, it is only recently that researchers have considered combining these two modalities of estimates on the same task^10^. Although the initial results appear promising, more efforts along this direction are needed to determine how the elicitation and aggregation of such multimodal inputs can further enhance WOC effects.

A quintessential example of a numerical estimation activity comes from the well-known experiment attributed to Galton^12^, in which county fair attendees were asked to guess the weight of an ox; the individual estimates were reported to be off the mark, but the aggregation of 787 guesses yielded a near-perfect estimate of the true value. Working with numerical estimates has led to WOC methods being linked most commonly with simple aggregation functions such as average, median, and mode^13^. The ease with which this type of estimates can be elicitated has also facilitated the wide deployment of WOC activities on crowdsourcing platforms (e.g., Amazon MTurk^14^, Prolific^15^). In turn, this has allowed researchers to experiment with a variety of novel treatments for further teasing out crowd wisdom. These include but are not limited to “debiasing training”^16^, conversational agents^17^, programmed micro-breaks^18^, and the “sharing of social information” provided by other participants—and even by computer models—during input elicitation^19,20^.

Numerical estimation activities have also been featured in the exploration of a WOC offshoot termed “wisdom of the inner crowd” (WOIC)^21,22^, which holds that better individual (and collective) estimates can be attained by eliciting and aggregating multiple estimates from each participant, such as eliciting estimates on the same numerosity question at varying time intervals (e.g.,^23–25^) and prompting participants to reconsider their initial estimates, known as “dialectical bootstrapping”^21^. Other work has demonstrated that eliciting responses to a logically equivalent rephrasing of the original question tends to outperform asking the same question on separate occasions^26,27^. Although these and other WOIC treatments can improve crowd wisdom, their complexity may make them difficult to implement outside of laboratory settings.

Ordering activities are less prominent in the literature, but they have also been employed to address a variety of complex tasks. Examples include arranging the 50 U.S. states from east to west, listing U.S. presidents based on when they served in office, and ordering international rivers from shortest to longest^8,9^. Ordering tasks may become more demanding because they require implicit pairwise comparisons between entities, whereas numerical estimation involves focusing on only one entity at a time. Ordering tasks also involve different types of aggregation methods. Among the most popular are voting rules designed to return an aggregate ranking that best represents the collection of input rankings according to social welfare functions, e.g., the Borda rule, the plurality rule, and the Copeland rule^8,28,29^. Certain functions are easy to evaluate, while others such as the Kemeny rule (see^30^) induce computationally intractable (i.e., NP-hard) problems and are usually modeled and solved using integer programming techniques—for example, a binary program developed for the Kemeny rule^31^ can be solved using the standard branch-and-bound algorithm. Probabilistic methods have also been applied, but they have performed consistently worse in this context^8^. In contrast with this diversity of aggregation methods, there has been little research on how different options for eliciting ordinal inputs may impact WOC and WOIC results.

In an effort to leverage multiple types of elicited inputs, Kemmer et al.^10^ devised a dot estimation study in which participants were asked both to order multiple images based on the number of dots they contain and to guess the respective number of dots in each image. The two types of estimates were aggregated using optimization models designed to return an aggregate ordinal-numerical pair that is closest to the collection of inputs according to social choice-inspired distance functions^32^. The collective orderings obtained in^10^ from these *multimodal aggregation models* consistently outperformed those obtained by aggregating the standalone ordinal inputs with traditional social welfare functions. Although these results intimate that using inputs of different modalities may enhance WOC effects, their generalizability is limited by their dependence on elaborate mathematical models and state-of-the-art optimization software. In fact, the best performing model entails solving an NP-hard problem. The aforementioned study also did not analyze the implications of different input elicitation options on WOC, let alone on individual accuracy.

This work analyzes in greater depth the degree to which the elicitation and aggregation of ordinal and numerical estimates can improve accuracy of individual and collective estimates on ordering tasks. To this end, it presents two intuitive elicitation options and deploys them via a crowdsourcing platform. Altogether, this work addresses three key research questions:

- Are there significantly different effects on individual and collective accuracy when ordinal and numerical inputs are elicited jointly versus independently?
- Can fast, commonly accessible aggregation methods leverage the two modalities of estimates to improve crowd wisdom effects?
- What are the added benefits of using the two modalities of estimates from each participant versus aggregating ordinal estimates from a group of participants and numerical estimates from a different group of participants?

## Results

**Figure 1:**
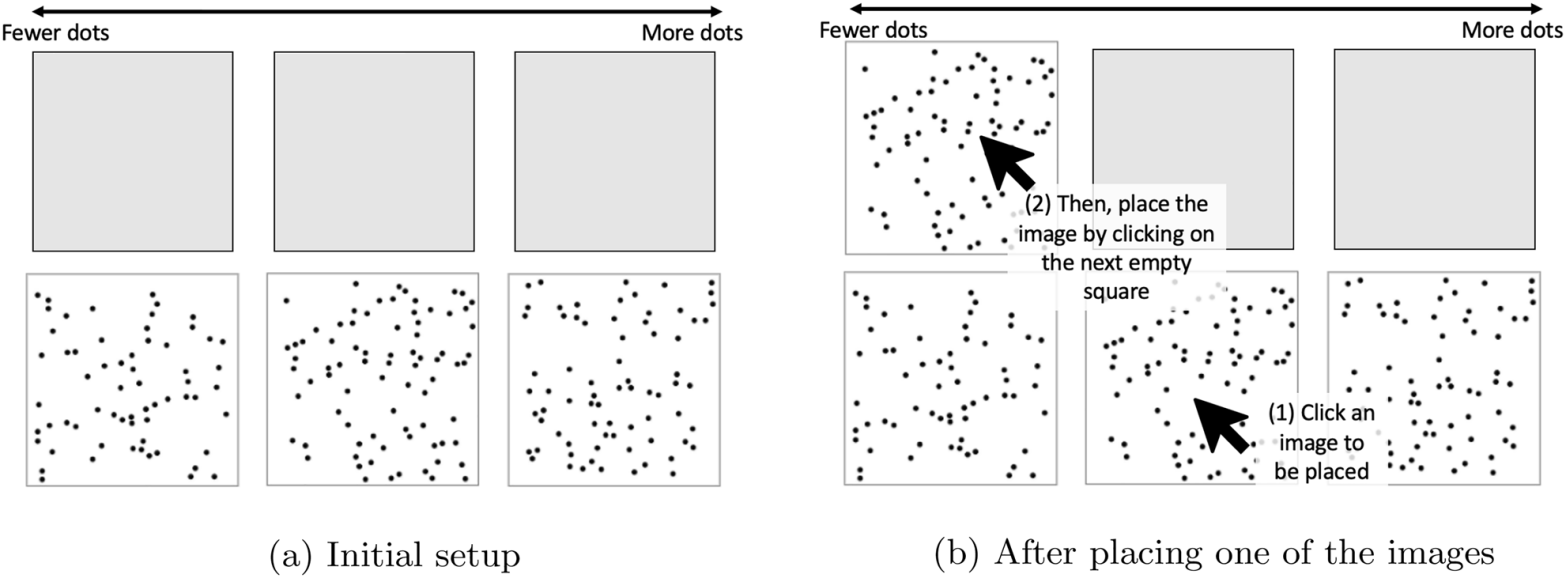
Ordinal elicitation interface for the 3-image task.

To address the research questions, we designed a web-based study via Amazon Mechanical Turk (*N*= 600) to crowdsource the ordering of different sets of images based on the number of dots contained in each image. There are 30 images in a set, with distinct quantities of black dots, ranging from 50 to 79, scattered randomly onto a white background. Each participant is assigned four smaller ordering tasks (i.e., image subsets) consisting of a pseudorandom selection of 2, 3, 5, and 6 images, respectively; there is a different 30-image dataset associated with each of the four task sizes. The specific subsets are assigned to participants so that all 30 images are viewed the same number of times per task (see Methods for more details). In the ordinal estimation task, the assigned images are provided along the bottom row of the interface. Participants click on each image and then on a blank square on the top row to arrange the images in increasing order of the number of dots they contain. For visual reference, Fig. 1 provides an example of the 3-image ordering task; Fig. 1a shows the initial setup (i.e., before the participant orders the images), and Fig. 1b shows the interface following the placement of the first image.

**Figure 2:**
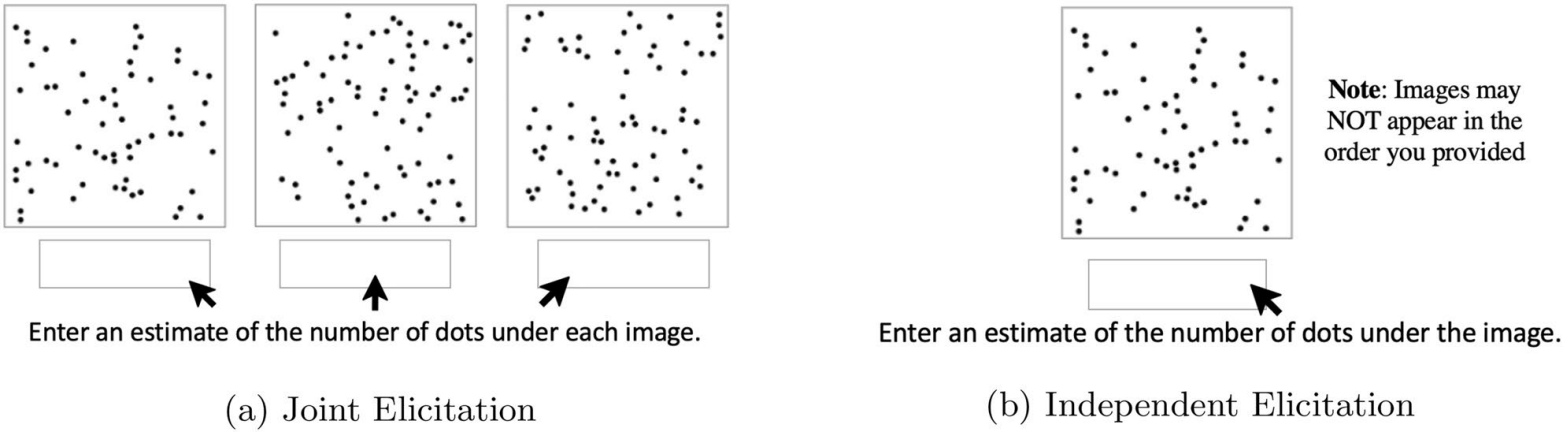
Numerical elicitation interfaces for the 3-image task.

Participants then proceed to the *numerical estimation task*, which entails guessing the total number of dots in each of the images they saw in the preceding ordering task. For half of the participants, this information is elicited *jointly*, that is, all assigned images are shown side-by-side as the participant arranged them in the ordering task with a text box underneath each image to elicit the numerical estimates (see Fig. 2a). For the other half of participants, numerical estimates are elicited *independently*; that is, each assigned image is shown individually and in a randomized order, with an input text box underneath (see Fig. 2b). Figure 2a and b show the interfaces of these two distinct numerical elicitation options, referred to as the *joint-elicitation interface* and the *independent-elicitation interface*, respectively. In effect, the joint-elicitation interface is designed to increase the efficiency and convenience of eliciting the two input modalities, while the independent-elicitation interface is intended to attenuate potential negative impacts of accessing one’s own earlier estimates^33^. Before proceeding, it is important to elaborate on a few additional details regarding the crowdsourced activity. First, Figs. 1 and 2 are stylized representations; screenshots of the actual interfaces seen by my participants are included in the Supplementary Material. Second, participants were compensated with a payment of $1.00 for approximately 5 minutes of work, irrespective of the accuracy of their estimates. Finally, the participant demographics are summarized in the Supplementary Material.

After the study was completed, the data are aggregated using methods that support both ordinal and numerical inputs. As a baseline of comparison, we employ the cardinal (i.e., numerical) and ordinal aggregation model (COA), which is the optimization-based method that achieved the best performance in^10^. As Sect. "Introduction" explains, COA represents an NP-hard optimization problem and thereby poses computational scalability issues. In pilot computational tests, individual problem instances associated with this study took up to 10 hours to solve using the mixed-integer programming techniques introduced in^32^. Hence, a 10-minute time limit is imposed per COA instance, after which the best incumbent solution is extracted, along with its *optimality gap*—that is, a provable worst-case bound on how far its objective function value could be from the optimal value. While some instances were solved to optimality, many were not. However, these optimality gaps varied between 0.01% and 11%, meaning that the performance of COA for any of the instances that was terminated after 10 minutes of solve time could be at best 11% better than what is reported in the results.

The remaining aggregation methods took fractions of a second to solve each problem instance. These are based on traditional voting rules that have performed well in previous ordinal estimation studies: Plurality voting, Borda Count, and Copeland’s method^30^. Each requires a set of ranking vectors as inputs and returns an aggregate ranking vector. They are extended to the context at hand through a simple data conversion. Specifically, the vector of numerical estimates of participant $\ell$, say $\varvec{b}^{\ell }$, is first converted into a ranking vector, denoted as rank$(\varvec{b}^{\ell })$; the *i*th element in the vector indicates the position that participant $\ell$’s numerical estimate for image *i* occupies within their full list of estimates, sorted in non-decreasing order and accounting for ties. Then, the voting rule is applied to aggregate the vectors rank$(\varvec{b}^{\ell })$ of all participants as well as their respective ordinal estimates, denoted as $\varvec{a}^{\ell }$. It is important to remark that the conversion into ordinal estimates truncates differences about the precision of the individual numerical estimates. This does not occur with the COA model, which imposes a different type of penalty to each modality of inputs (see^32^ for more details).

### Individual performance

**Figure 3:**
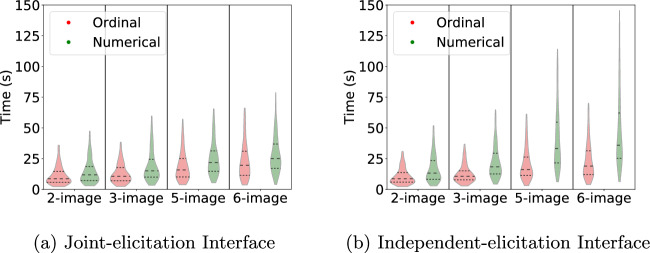
Completion times by interface, estimation task, and task size.

To highlight the basic differences between the elicitation interfaces, we first delve into two aspects at the individual participant level: completion time and degree of self-contradiction. Figure 3 summarizes the completion time for each of the four task sizes and the two interfaces. Outliers are excluded to improve analysis and visualization. They were identified based on standard statistical criteria: data points below $Q1 - 1.5 \times IQR$ and above $Q3 + 1.5 \times IQR$, with *IQR* representing the interquartile range calculated as $Q3 - Q1$ (where *Q*1 is the first quartile, and *Q*3 is the third quartile).

To assess whether the differences in completion times between numerical estimation and ordinal estimation tasks are statistically significant, we conducted a two-sample *t*-test on the average completion times for each of the four task sizes and the two interfaces (see the Supplementary Material for the detailed results). With the independent-elicitation interface, the average differences between the ordinal and the numerical estimation task completion times were 8.12, 15.28, 27.54, and 38.99 seconds for the 2-, 3-, 5-, and 6-image tasks, respectively; with the joint-elicitation interface, the corresponding average differences were 4.86, 15.42, 5.49, and 14.49 seconds. In short, the numerical estimation task took significantly longer than the ordinal estimation task in the 3-image and 6-image tasks with the joint-elicitation interface; the same relationship held true for the 3-, 5-, and 6-image tasks with the independent-elicitation interface (see the Supplementary Material for the specific *p*-values).

Next, we delve into the differences in completion times between the two interfaces. Similarly, we conducted a two-sample *t*-test to determine whether the average completion times of each task in the joint-elicitation interface and in the independent-elicitation interface are statistically significantly different. Ordinal task completion times were relatively indistinguishable across the two interfaces, which can be determined by comparing one-by-one and side-by-side the red violin plots in Fig. 3a and b corresponding to the results shown in Appendix Tables 2 and 3 in the Supplementary Material. On the other hand, the numerical estimation completion times were longer with the independent-elicitation interface for the larger task sizes, specifically, they were 21.17 and 27.58 seconds higher on average than with the joint-elicitation interface for the 5- and 6-image tasks, respectively. These noticeably longer completion times indicate that numerical estimates are more easily elicited with the joint-elicitation interface, as expected. On the other hand, average differences in numerical estimation completion times between the two interfaces were not as significant for the two smaller task sizes—the independent-elicitation interface took 4.50 seconds more for the 2-image task but 0.47 seconds less on the 3-image task. One plausible explanation is that it is less cognitively demanding (although not necessarily conducive to more accurate results) to provide a numerical guess based on a prior ordering estimate. However, for smaller-sized tasks, having such a point of reference does not seem to yield a significant advantage.

**Figure 4:**
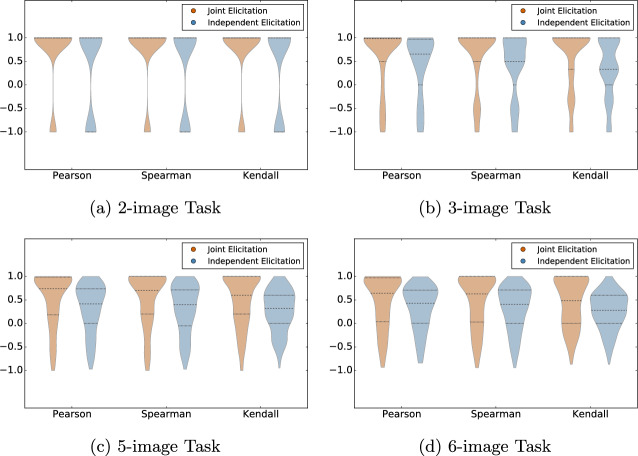
Correlation between ordinal and numerical estimates for each elicitation interface.

To report on the degree of the self-contradiction of the multimodal input estimates, we analyze the similarity between $\varvec{a}^{\ell }$ and rank($\varvec{b}^{\ell }$), for each participant ${\ell }$. Their similarity is measured using three correlation coefficients, namely Kendall-$\tau$, Spearmans’ $\rho$, and Pearson, which have been previously employed for related purposes (e.g., see^34–36^). The domain of each measure is $[-1, 1]$; the left and right endpoints connote complete negative and positive correlation, respectively, and the midpoint 0 connotes no association. As shown in Fig. 4, participants’ ordinal estimates were more similar to (the orderings induced by) their numerical estimates when the two modalities of input were elicited jointly. For instance, the median Kendall-$\tau$ correlations were 1.0, 1.0, 0.6, 0.47 with the joint-elicitation interface and 1.0, 0.33, 0.31, and 0.28 with the independent-elicitation interface, for the 2-, 3-, 5-, and 6-image tasks, respectively; correlation values varied with the other two coefficients, but similar discrepancies across the interfaces can be observed. These statistics reflect that having access to one’s own ordinal estimates can help maintain relative consistency in one’s numerical estimates.

**Figure 5:**
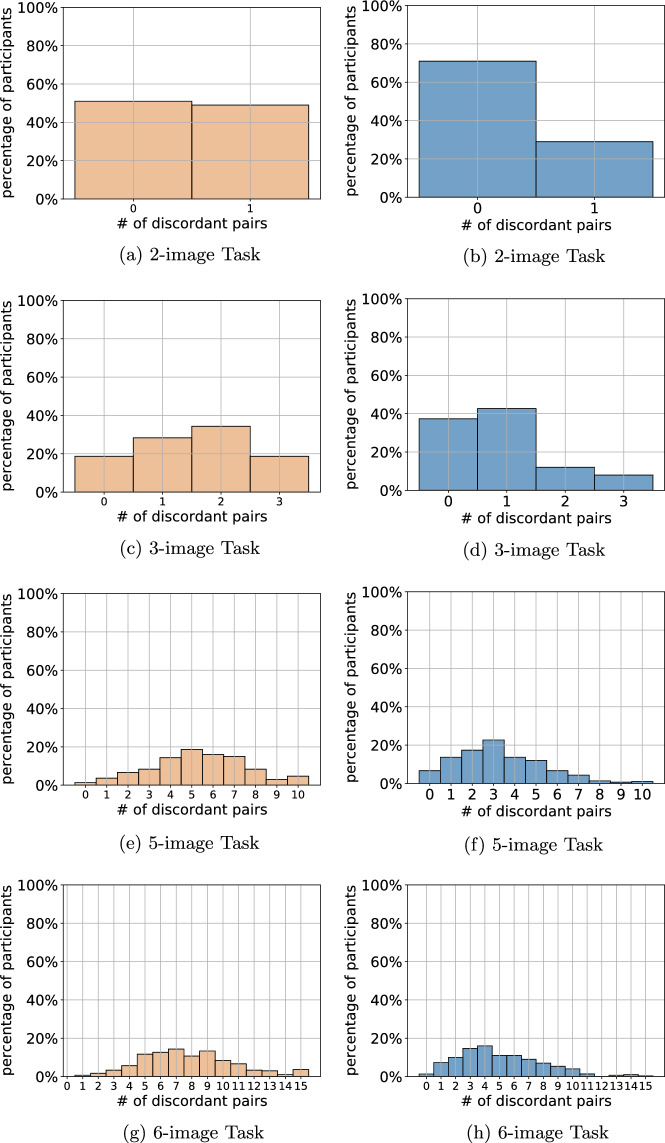
Distribution of individual ordinal estimation errors resulting from the (converted) numerical estimation inputs (left column: joint-elicitation interface, right column: independent-elicitation interface).

Next, we analyze how participants performed individually at estimating the correct ordering of the image subsets assigned to them. Individual performance on the ordinal estimation task was relatively indistinguishable for the two participant groups, because this interface was identical for all participants. Hence, the analysis focuses on the individual ordinal estimates induced from each participant’s inputs in the numerical estimation task (i.e., rank($\varvec{b}^{\ell }$)). The converted inputs are compared to the ground-truth ordering of the respective image subset seen by a participant by counting their *discordant pairs*, that is, the total pairs of images where the participant’s ordering is the reverse of the true ordering. A perfectly ordered subset of images yields no discordant pairs, and less accurate orderings result in more discordant pairs. When ordering *n* images, the maximum number of discordant pairs is $n(n-1)/2$; hence, there can be at most 1, 3, 10, and 15 discordant pairs, corresponding to the last tick on the *x*-axis in Fig. 5, for the 2-, 3-, 5-, and 6-image tasks, respectively.

Figure 5 plots individual accuracy for each of the four task sizes and two interfaces via bar graphs; each represents the percentage of participants who attained one of the possible numbers of discordant pairs, which are enumerated along the *x*-axis. For instance, over 50% of the joint-elicitation interface participants submitted numerical estimates that induced a perfect ordering for the 2-image task (see Fig. 5a) but less than 1% did so for the 6-image task (see Fig. 5g). A similar pattern of decreasing accuracy is evident in the independent-elicitation interface.

Figure 5 also demonstrates that the inputs gathered via the joint-elicitation interface resulted in lower-quality estimates. For example, the proportion of perfect orderings in the 2-image task was higher for the independent-elicitation interface by approximately 20 percentage points. More generally, for each of the four task sizes, the distribution associated with the independent-elicitation interface skews right (i.e., more mass is concentrated on the *x*-values that connote fewer errors), with a roughly descending pattern as one moves right. On the other hand, those associated with the joint-elicitation interface increasingly resemble normal distributions, with the values concentrated toward the middle. These results indicate that, although eliciting multiple modalities of estimates jointly led to less self-contradiction, this had a negative effect on individual accuracy. A natural explanation is that with this method of elicitation, errors in the ordering task are less likely to be self-corrected through the numerical estimation task.

### Crowd performance

**Figure 6:**
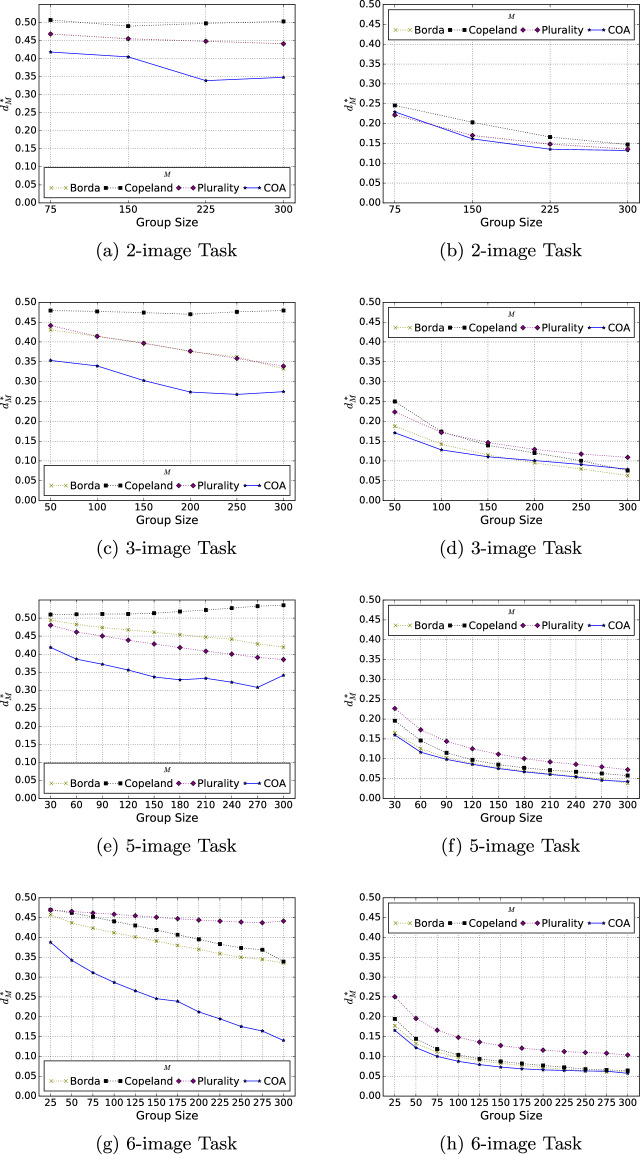
Accuracy of collective ordinal estimates - lower values of $d^*_M$ connote more accurate collective estimates (left column: joint-elicitation interface, right column: independent-elicitation interface).

Crowd performance is calculated by aggregating the participants’ estimates using the methods described in Sect. “Aggregation Methods” Section. The results are reported in Fig. 6 via eight two-dimensional plots corresponding to each possible combination of task size and interface. In each plot, the *x*-axis indicates the group size, which is regulated by taking subsets of the input data (refer to Sect. “Notes on Task Allocation” for more details). The *y*-axis reports the Kemeny-Snell distance^37^ (i.e., the number of discordant pairs) between each collective estimate and the ground truth. It is henceforth denoted as $d^*_M$, where *M* represents the aggregation method used to obtain the collective estimate. The reported metric has been normalized to lie between 0 and 1, both inclusive, by dividing by the maximum number of pairwise reversals between two rankings of each respective size^38^. Note that lower values of $d^*_M$ correspond to more accurate collective estimates (since this metric reflects the distance or disagreement with the ground truth).

The results support the wisdom of crowds effect, i.e., that accuracy tends to improve as more individual estimates are aggregated. This holds for both interfaces and across all tested task sizes and aggregation methods. However, the collective estimates associated with the independent-elicitation interface significantly outperformed those associated with the joint-elicitation interface; in fact, nearly every collective estimate obtained with the independent-elicitation interface is more accurate than the best collective estimate obtained with the joint-elicitation interface (in Fig. 6, nearly every point on a specific-color curve in the right column is located below the same-colored curve in the left column). For example, in the 5-image task, $d^*_{COA}=0.16$ when using a group of 30 participants with the independent-elicitation interface, whereas $d^*_{COA}=0.34$ when using ten times the number of participants with the joint-elicitation interface. Similar performance disparities occurred with other task sizes and aggregation methods demonstrating that, although WOC effects are observed in both interfaces, eliciting numerical estimates independently from ordinal estimates resulted in more accurate collective estimations. Based on these findings and those of Sect. "Individual performance" the remainder of this paper will focus on the inputs obtained with the independent-elicitation interface.

Next, we compare the quality of the collective estimates attained with the tested aggregation methods. The optimization-based method (the COA model) consistently achieved better results than the voting rule-based methods, as shown in Fig. 6. Specifically, for the joint-elicitation interface, the COA model outperformed the rest of the methods by an average distance from the ground truth ($d^*_M$, for each respective method *M*) of 0.14, with variations ranging from as little as 0.04 to as large as 0.30. Although the differences between the optimization-based method and the voting rule-based methods diminish in the independent-elicitation interface, the COA model still edged out voting rule-based methods in most cases by an average $d^*_M$ value of 0.02, with variations as large as 0.08. It is worth reiterating that the COA model did not reach optimality at the ten-minute time limit in many cases; however, based on the recorded optimality gaps, which were 10.89% or lower on every instance, its performance is not expected to be dramatically better. Its performance edge was highest when fewer inputs were used to derive the collective estimate. This highlights a significant practical advantage of this method, as the cost of crowdsourcing increases with the number of participants recruited. However, COA is also the method that requires the highest computational effort. In fact, for most of the instances generated from this study and especially for the higher task sizes (5-image and 6-image tasks), it exceeded the 10-minute time limit. Moreover, as more individual estimates are aggregated, most of the multimodal voting rules exhibit a comparable performance to COA. Among them, the multimodal Borda rule attained the best results, and it even outperformed COA in some cases in the independent-elicitation interface—specifically, with inputs from 300 participants in the 3-image task, $d^*_{Borda}=0.06$ and $d^*_{COA}=0.08$, and with inputs from 300 participants in the 5-image task, $d^*_{Borda}=0.038$ and $d^*_{COA}=0.043$. Note that all of the voting-based methods required less than one second of computation time. Altogether, these comparisons demonstrate that, given sufficient individual estimates, high-quality collective estimates can be obtained with simpler aggregation methods and nominal computational effort.

### Assessing the added value of multimodal elicitation

This subsection explores further how multimodal estimates can affect crowd wisdom. In particular, we compare the effectiveness of aggregating the ordinal and numerical estimates from a single group of individuals (“coupled-estimate group”) against mixing the ordinal estimates from one group of individuals with the numerical estimates from a different group (“separate-estimate groups”) and then aggregating the two modalities of estimates together. Based on the findings of Sect. "Crowd performance", the multimodal Borda voting rule is used to aggregate the estimates in both cases, to take advantage of its computational ease and high-quality outputs.

**Figure 7:**
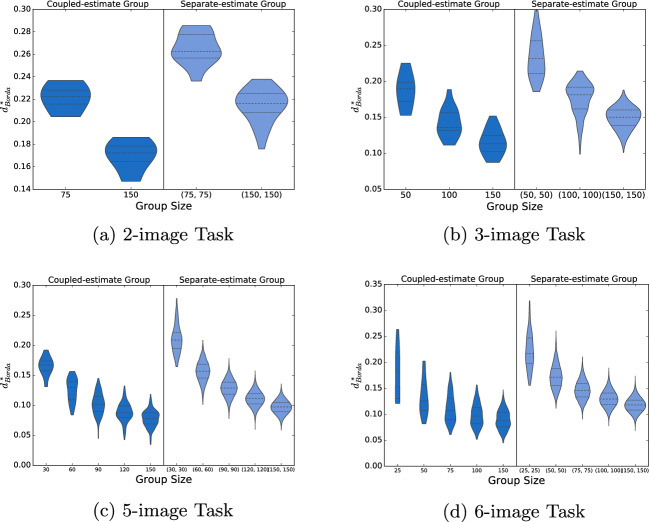
Accuracy of collective estimates obtained from coupled-estimate groups and separate-estimate groups of different sizes (lower $d^*_{Borda}$ values connote more accurate collective estimates).

Figure 7 contains one plot for each of the four task sizes comparing the performances of the coupled-estimate groups (left of the dividing line) and of the separate-estimate groups (right of the dividing line). The *x*-axis displays the group size; it is reported as a single number for the coupled-estimate group and as an ordered pair for the separate-estimate group, with the first coordinate indicating the size of the group providing ordinal estimates and the second the size of the group providing numerical estimates. The *y*-axis provides the respective $d^*_{Borda}$ distance (lower values connote more accurate collective estimates).

The four subfigures show that smaller coupled-estimate groups perform on par or better than larger separate-estimate groups. For example, in the 3-image task, coupled-estimate groups of size 100 yielded a more accurate collective estimate (median distance of 0.14) than separate-estimate groups of size (100, 100) (median distance of 0.18); note that the total number of estimates is identical in both cases, but the separate-estimate group has twice the number of participants. More impressively, in the 3-image task, coupled-estimate groups of size 100 performed comparably to separate-estimate groups of size (150, 150), with median $d^*_{Borda}$ distances of 0.14 and 0.15, respectively, and virtually identical minimum and maximum values. In this case, the separate-estimate groups entailed 200% more participants and 50% more total inputs.

It is worth emphasizing that, although an equal number of ordinal inputs and numerical inputs are aggregated to obtain each of the collective estimates plotted in Fig. 7, the coupled-estimate groups’ estimates benefit from having their two modalities of inputs come from the same set of participants. In effect, two pieces of interrelated information from each image in an assigned subset are used in the coupled-estimate groups’ estimates, while only one piece of information from each image in an assigned subset is used in the separate-estimate groups’ estimates—although the latter use information from twice the number of combinations of image subsets. Based on these observations, we conjecture that the main mechanism underlying this result is linked with the concept of WOIC (see Sect. "Introduction"). In fact, the coupled-estimate groups’ results may additionally benefit from using the participants’ multiple responses from logically equivalent rephrasing of a given question (i.e., ordering and numerical estimates for each assigned image)^26,27^.

These differing performances support the notion that, for ordering tasks, the two elicited estimate modalities possess complementary information that can help to counteract each individual’s errors from completing ordinal or numerical estimation tasks. In other words, when data from only one estimate modality is utilized from each participant, this complementarity is lost. Based on this explanation, another mechanism that could be potentially at work is cognitive-process diversity^39^, which suggests that the aggregation of several estimates is enhanced when the estimates are based on different cognitive processes. Grounded on dual process theory, cognitive-process diversity differentiates between *intuitive* processes, characterized by being preconscious and fast and *analytical* processes, characterized as slow, deliberative, rule-governed, and conscious^40^. The ordinal estimation task fits the description of an analytical process; the numerical estimation task could fit the description of either of the two cognitive processes, depending on the specific response times and frame of mind of each participant. However, this statement is based on speculation and needs to be rigorously tested with a specialized experimental design. As such, it is left for future work.

## Discussion

This study contributes to the understanding of how joint multimodal input elicitation and aggregation can enhance wisdom of crowds effects^1^ on ordering tasks. Firstly, we show that the input elicitation modality affects the quality of information and any subsequent aggregation thereof. Participants who provided numerical estimates independently from their ordinal estimates over a subset of images with dots (i.e., those who received the independent-elicitation interface) provided considerably better estimates than those who completed them jointly (i.e., those who received the joint-elicitation interface). Caveats for the higher level of accuracy attained with the separate-elicitation interface are that participants required relatively more time to provide numerical estimates for each image in their assigned subset and that their multimodal inputs were more self-contradictory. Nonetheless, the latter finding aligns with a core principle of WOC, namely that erroneous inputs can cancel out to produce better collective estimates.

Secondly, we demonstrate that the enhanced WOC effects from multimodal estimates documented herein are not reserved for optimization-based methods. They are also accessible to commonly used voting rules, following a suitable data conversion relevant to ordering tasks. Indeed, although the optimization model produced better collective estimates when relatively few inputs were available, the modified voting rules yielded nearly the same level of accuracy in fractions of a second, and they entailed a few lines of code reproducible with desktop programs. These findings help to overcome some of the impractical aspects of aggregating multimodal estimates using optimization-based methods^10,32^, namely, the need for specialized software and prohibitive computational times—which took as long as 10 hours for some of the instances generated from this study.

Thirdly, the featured computational experiments highlight the added value of jointly eliciting multiple modalities of estimates from a single group of participants, as opposed to mixing the inputs from two groups who each provide estimates of a single but distinct modality. The former tends to yield better collective estimates even when there is an equal or higher number of total estimates aggregated from the latter. Stated otherwise, the single group joint estimation design enables the extraction of higher-quality estimates with fewer resources. These findings have important implications for the design of cost-effective crowdsourcing activities and surveys, where recruitment costs and time can be limiting factors.

Fourthly, another contribution relates to the burgeoning concept of the wisdom of crowds in one mind^21,22^, which holds that better individual (and collective) estimates can be attained by gathering and aggregating multiple estimates from each participant in the crowd. Prior works have lent support to this phenomenon mostly by eliciting estimates regarding the same question at varying time intervals (e.g., see^23–25^). More advanced implementations have demonstrated that eliciting responses to a logically equivalent rephrasing of the original question outperforms asking the same question on separate occasions^26,27^. The multimodal input elicitation method featured herein further supports this idea, with the added contribution of allowing two distinct, logically interrelated questions to be asked of each image from each participant over a single sitting. The takeaways from this analysis further demonstrate that certain choices in how multimodal estimates are obtained and used can have different practical implications. In particular, using multiple modalities of estimates from each participant can help extract WOC effects from smaller groups relative to mixing the numerical estimates of one group with the ordinal estimates from a different group. If these findings can be replicated and scaled to more ordering tasks, they could help reduce costs, especially in situations in which recruiting more participants is costlier than increasing individual workload. It is also relevant to remark that the presented method of eliciting the multiple modalities of estimates independently rather than jointly aligns with the wisdom of the inner crowd guidance to design questions that can generate estimates with statistically independent errors^22,41^.

The outcomes of this research evoke compelling questions for further research. To begin, the presented study used numerical estimates to complement and enhance collective ordinal estimates on ordering tasks, but it did not delve into the converse, that is, whether and how ordinal estimates can be leveraged for numerosity tasks. Moreover, the featured tasks entailed human computation activities with a known ground truth—i.e., the ordering of the image sets is known a priori. On many real-world tasks that harness the power of crowdsourcing and collective intelligence, a ground truth may not be immediately available—e.g., stock prediction^42^—or it may not exist altogether—e.g., crowdsourced opinion^43^. Hence, an interesting research direction is to explore whether the proposed methods would be beneficial in those contexts as well. Finally, the practical benefits of multimodal aggregation motivate theoretical questions beyond wisdom of crowds, including the sociotheoretic analysis of voting rules (see^30^) that accept both numerical and ordinal inputs and the study of the polyhedral structure (see^44^) underlying the optimization model.

## Methods

### Distance functions

The optimization-based method (the COA model^32^) finds a pair of ordinal and numerical estimate vectors that minimize the cumulative disagreement with the individual input estimates, according to two suitable distance functions. The distance functions used in this study are the Normalized Projected Kemeny-Snell distance ($d_{NPKS}$)^38^ and the Normalized Project Cook-Kress distance ($d_{NPCK}$)^45^, respectively; these are extensions of the Kemeny-Snell distance ($d_{KS}$)^37^ and the Cook-Kress distance ($d_{CK}$)^46^). The mathematical descriptions are provided in the ensuing paragraphs.

To define the distance functions, it is important to introduce the requisite notation. Denote *V* as the full set of alternatives (i.e., universal set of images) to be evaluated. Let $\varvec{a}^{\ell }$ and $\varvec{b}^{\ell }$ denote the ordinal and numerical estimate vectors, respectively, gathered from participant $\ell \in L$. The subset of alternatives evaluated in $\varvec{a}$ (resp., $\varvec{b}$) is denoted as $V_{\varvec{a}}$ (resp., $V_{\varvec{b}}$); then $a^{\ell }_i$ (resp., $b^{\ell }_i$) denotes the rank position (resp., numerical guess) provided by participant $\ell$ for alternative $i\in V_{\varvec{a}}$ (resp., $i\in V_{\varvec{b}}$). Finally, ${V_{\varvec{a}^1}}\bigcap {V_{\varvec{a}^2}}$ represents the subset of alternatives evaluated in both rankings $\varvec{a}^1$ and $\varvec{a}^2$, and ${\varvec{a}^1}_{({V_{\varvec{a}^1}}\!\cap {V_{\varvec{a}^2}})}$ and ${\varvec{a}^2}_{({V_{\varvec{a}^1}}\!\cap {V_{\varvec{a}^2}})}$ denote the projections of each ranking onto the subset of alternatives evaluated in both rankings (the analogous definition of the common subset of alternatives evaluated in both $\varvec{b}^1$ and $\varvec{b}^2$ is omitted for simplicity).

The distance function $d_{NPKS}$ between two possibly incomplete rankings is defined as^38^:

$$\begin{aligned} d_{NPKS}(\varvec{a}^1,\varvec{a}^2)= {\left\{ \begin{array}{ll} \frac{d_{KS}(\varvec{a}^1\vert _{({V_{\varvec{a}^1}}\cap {V_{\varvec{a}^2}})},\varvec{a}^2\vert _{({V_{\varvec{a}^1}}\cap {V_{\varvec{a}^2}})})}{\left|{V_{\varvec{a}^1}}\bigcap {V_{\varvec{a}^2}}\right|(\left|{V_{\varvec{a}^1}}\bigcap {V_{\varvec{a}^2}}\right|-1)/2} &{} \text {if } \left|{V_{\varvec{a}^1}}\bigcap {V_{\varvec{a}^2}}\right|\ge 2,\\ 0 &{} \text {otherwise}. \end{array}\right. } \end{aligned}$$

Here, $d_{KS}$ is a distance metric that counts the number of discordant pairs between two complete rankings $\varvec{a}^1$ and $\varvec{a}^2$ over *n* alternatives and is defined as^37^:

$$\begin{aligned} d_{KS}(\varvec{a}^1,\varvec{a}^2) = \frac{1}{2} \sum _{i=1}^n\sum _{j=1}^n\left|\text {sign}(a^1_i-a^1_j)-\text {sign}(a^2_i-a^2_j)\right|. \end{aligned}$$

The distance function $d_{NPKS}$ is nonlinear. The scaled Kendall tau-extended correlation coefficient^47^ is an alternative linear measure on incomplete rankings that can be equivalently used via a binary programming formulation to solve the Kemeny ranking aggregation problem^31^.

The distance function $d_{NPCK}$ between two possibly incomplete numerical estimation vectors is defined as^45^:

$$\begin{aligned} d_{NPCK}(\varvec{b}^1,\varvec{b}^2)&= {\left\{ \begin{array}{ll} \frac{d_{CK}(\varvec{b}^1\vert _{({V_{\varvec{b}^1}}\cap {V_{\varvec{b}^2}})},\varvec{b}^2\vert _{({V_{\varvec{b}^1}}\cap {V_{\varvec{b}^2}})})}{4R \cdot \big \lceil \frac{\left|{V_{\varvec{b}^1}}\!\cap {V_{\varvec{b}^2}}\right|}{2}\big \rceil \cdot \big \lfloor \frac{\left|{V_{\varvec{b}^1}}\!\cap {V_{\varvec{b}^2}}\right|}{2}\big \rfloor } &{} \text {if } \left|{V_{\varvec{b}^1}}\bigcap {V_{\varvec{b}^2}}\right| \ge 2,\\ 0 &{} \text {otherwise}; \end{array}\right. } \end{aligned}$$

where where $R:=U-L$ denotes the range of the numerical estimates (*U* is the highest value and *L* the lowest value in the inputs). Here, $d_{CK}$ is a distance metric that calculates the pairwise differences of intensity between two complete numerical estimation vectors $\varvec{b}^1$ and $\varvec{b}^2$ over *n* alternatives and is defined as^46^:

$$\begin{aligned} d_{CK}(\varvec{b}^1,\varvec{b}^2) = \frac{1}{2}\sum _{i=1}^n\sum _{j=1}^n\left|(b^1_i-b^1_j)-(b^2_i-b^2_j)\right|. \end{aligned}$$

### Aggregation methods

This section introduces the aggregation methods employed in the paper. First, it presents the optimization-based method, which builds on the distance functions described in the preceding subsection. Then, it presents three traditional voting rule-based methods defined for ordinal vectors, and it describes how they are extended to handle numerical estimate vectors.

#### Optimization-based method: the cardinal and ordinal aggregation (COA) model

The COA model jointly aggregates a set of cardinal (i.e., numerical) and ordinal vectors using distance functions $d_{NPKS}$ and $d_{NPCK}$. The optimization model finds the cardinal-ordinal vector pair $(\varvec{r}^*, \text {rank}(\varvec{r}^*))$ that minimizes the cumulative distance to the given inputs; its definition can be abbreviated as:

$$\begin{aligned} \underset{\varvec{r}}{\min }\ \sum _{\ell =1}^{\left|L\right|} \text { }d_{NPCK} (\varvec{b}^{\ell }, \varvec{r}) + \sum _{\ell =1}^{\left|L\right|} \text { }d_{NPKS} (\varvec{a}^{\ell }, \text {rank}(\varvec{r})). \end{aligned}$$

In the solution, the ordinal vector $\text {rank}(\varvec{r})$ is induced by ordering the numerical values in $\varvec{r}$. This logic is enforced through linearized expressions (see^32^), which build on the binary programming formulation for the Kemeny ranking aggregation problem^31^.

#### Voting rule-based methods

The three voting rules featured in this study are applicable to ordinal vectors (i.e., rankings). In social choice theory, they are formally denoted as *social choice functions*, which map a given set of preference rankings over a set of alternatives into a winner, meaning that they return a single alternative rather than a ranking of all of the alternatives^30^. To obtain a full ranking (or *social ordering*), all alternatives are ordered in non-increasing order of the respective scores assigned by the voting rule. Moreover, each rule is extended to handle numerical estimate vectors through a simple data conversion. Specifically, the vector of numerical estimates of participant $\ell$, $\varvec{b}^{\ell }$, is first converted into a ranking vector, denoted as rank$(\varvec{b}^{\ell })$; the *i*th element in the vector indicates the position that participant $\ell$’s numerical estimate for alternative *i* occupies within their full list of estimates, sorted in non-decreasing order and accounting for ties. The mathematical description of the voting rules is provided in the ensuing paragraphs.

**The plurality rule** selects the alternative with the most first-place votes. The function for determining whether alternative *i* is in first place in the ordinal vector from participant $\ell \in L$ is given by^30^:

$$\begin{aligned} f(a^{\ell }_i)= {\left\{ \begin{array}{ll} 1 &{} \text{ if } a^{\ell }_i = 1,\\ 0 &{} \text {else}. \end{array}\right. } \end{aligned}$$

The plurality rule assigns a score to each alternative based on its number of first-place votes; the score of alternative *i* can be obtained as:

$$\begin{aligned} \text {plurality}(i) = \sum _{\ell =1}^{\left|L\right|} f(a^{\ell }_i). \end{aligned}$$

**The Borda rule** assigns a score to each alternative in a ballot according to how many alternatives it defeats, and it chooses the alternative with the highest score as the winner^30^. Mathematically, assuming that there exist *n* alternatives and the highest score is $n-1$ (i.e., there are at most $n-1$ alternatives ranked lower than the first-placed alternative), the Borda rule assigns a score to each alternative, where the score of alternative *i* is defined as:

$$\begin{aligned} \text {Borda}(i) = \sum _{\ell =1}^{\left|L\right|} (n - a^{\ell }_i). \end{aligned}$$

**The Copeland rule** chooses the alternative with the highest number of pairwise wins minus defeats as a winner, which is mathematically written as^30^:

$$\begin{aligned} \text {Copeland}(i) = \sum _{j\in V, j\ne i} \sum _{\ell =1}^{\left|L\right|} (\left|\{\varvec{a}^{\ell }:a^{\ell }_i < a^{\ell }_j\}\right|-\left|\{\varvec{a}^{\ell }: a^{\ell }_i > a^{\ell }_j\}\right|). \end{aligned}$$

### Notes on task allocation

To gain a better understanding of the plots in Fig. 6, which showcase the quality of collective estimates across various group sizes, it is useful to elaborate on how the image subsets were divided and allocated. For each task size $x\in \{2,3,5,6\}$, the image subsets assigned to each participant were generated by first randomly permuting the integers from 1 to 30 and then assigning the images corresponding to the first *x* integers to the first participant, the images corresponding to the second *x* integers to the second participant, and so on, until all 30 integers are allocated; this is repeated with a new random permutation of the integers from 1 to 30, as needed. Notice that 30 is exactly divisible by 2, 3, 5, and 6, meaning that the full set of 30 images randomized with each permutation can be allocated without remainder.

It is also helpful to specify how many times each image is evaluated based on the corresponding group size. As previously mentioned, a total of 300 participants took part in the study, and the number of times each image was shown varied depending on the task size and the subset of participants selected. For instance, in the 6-image task, with all 300 participants viewing 6 images each, there were a total of 1800 $(=300 \times 6)$ observations. Considering that there were 30 images in total, each image was viewed 60 ($=300 \times 6/30$) times (based on the aforementioned task allocation design). Similarly, in the 5-image task, with all 300 participants viewing 5 images each, there were 1500 $(=300 \times 5/30)$ observations, resulting in each image being seen 50 times ($=300 \times 5/30$). The calculation of how many times each image is seen can be generalized to different group sizes with the formula:

$$\begin{aligned} \frac{\text {(group size)} \times \text {(task size)}}{\text {total number of images}}. \end{aligned}$$

The formula can be applied when fewer participant estimates are used to obtain an aggregate estimate (i.e., to generate the collective estimates of smaller group sizes). For example, in the 2-image task, when estimates from 75 people are used (the left-most *x*-axis tick value), each image is seen only 5 times $(=75 \times 2/30)$.

### Human subjects study protocols

This research study involving human participants was designed and implemented according to Institutional Review Board (IRB) guidelines. The study was reviewed and approved by the Institutional IRB Committee within the Office of Research Integrity and Assurance at Arizona State University (STUDY00010770: “Estimating the number of dots in an image”). Accordingly, we affirm that (1) all methods employed were executed in strict accordance with pertinent regulations governing the research; (2) the experimental protocols received explicit approval before beyond deployed, ensuring adherence to ethical and regulatory standards; (3) informed consent was diligently obtained from all study subjects, without exception; and (4) participants were monetarily compensated.

### Supplementary Information

Supplementary Information.

## Data Availability

The datasets used and/or analyzed during the current study are available in the Github repository, https://github.com/ryankemmer/simpleRatingRanking.
