# Supplementary material for: Elicitation and aggregation of multimodal estimates improve wisdom of crowd effects on ordering tasks
Source: Sci Rep. 2024 Feb 1;14:2640. doi: 10.1038/s41598-024-52176-3 (PMC10834972; doi:10.1038/s41598-024-52176-3)
Supplement: Supplementary file 1 — Supplementary Information. [file 41598_2024_52176_MOESM1_ESM.pdf]

# Supplementary Materials

## Participants' Demographics

Appendix Table 1 provides the demographics of the study participants.

| | A | | B | |
|---|---|---|---|---|
| **Total** | 300 | | 300 | |
| **Gender** | | | | |
| Women | 78 | 26.00% | 116 | 38.67% |
| Men | 195 | 65.00% | 168 | 56.00% |
| Non-binary | 0 | 0.00% | 2 | 0.67% |
| Not answered | 27 | 9.00% | 14 | 4.66% |
| **Age** | | | | |
| 10-19 | 0 | 0.00% | 2 | 0.67% |
| 20-29 | 81 | 27.00% | 95 | 31.66% |
| 30-39 | 122 | 40.67% | 89 | 29.67% |
| 40-49 | 35 | 11.67% | 54 | 18.00% |
| 50-59 | 24 | 8.00% | 35 | 11.67% |
| 60-69 | 11 | 3.66% | 9 | 3.00% |
| 70-79 | 0 | 0.00% | 2 | 0.67% |
| Not answered | 27 | 9.00% | 14 | 4.66% |
| **Education** | | | | |
| 2-year degree | 12 | 4.00% | 29 | 9.67% |
| 4-year degree | 177 | 59.00% | 124 | 41.33% |
| College | 22 | 7.33% | 51 | 17.00% |
| Master's | 45 | 15.00% | 32 | 10.67% |
| Professional (MD, JD, etc) | 2 | 0.67% | 5 | 1.67% |
| Doctoral | 0 | 0.00% | 1 | 0.33% |
| High-school/GED | 15 | 5.00% | 43 | 14.33% |
| Less than high-school | 0 | 0.00% | 1 | 0.33% |
| Not answered | 27 | 9.00% | 14 | 4.67% |
| **Employment status** | | | | |
| Employed | 253 | 84.33% | 234 | 78.00% |
| Unemployed | 20 | 6.67% | 52 | 17.33% |
| Not answered | 27 | 9.00% | 14 | 4.67% |
| **Native English Speaker** | | | | |
| Native | 271 | 90.33% | 284 | 94.66% |
| Non-native | 2 | 0.67% | 2 | 0.67% |
| Not answered | 27 | 9.00% | 14 | 4.67% |

Appendix Table 1: Demographic Survey Results

## Elicitation Interfaces

The figures below illustrate the original, unaltered interfaces provided to study participants, representing non-simplified versions of Fig. 1 and Fig. 2 in the manuscript.

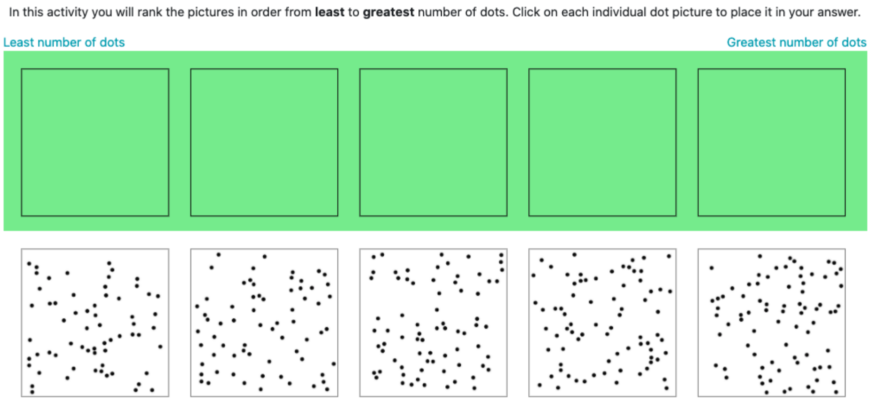


Appendix Figure 1: Ordinal Elicitation Interface for the 5-image Task

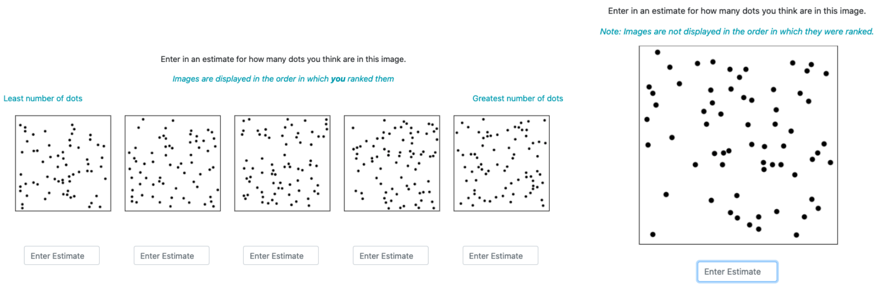


(a) Joint elicitation

(b) Independent elicitation (1 of 5 subtasks)

Appendix Figure 2: Numerical Elicitation Interface for the 5-image Task

## Task Completion Time

Appendix Table 2 and Table 3 display task completion times across different interfaces and task sizes, along with the corresponding test statistics and $p$-values.

Appendix Table 2: Completion Times with the Joint-elicitation Interface

| | Ordinal | Numerical | | |
|---|---|---|---|---|
| Task Size | Average (Standard Deviation) | Average (Standard Deviation) | $t$-statistic | $p$-value |
| 2-image | 17.42 (23.97) | 22.28 (36.00) | -1.94 | 0.052 |
| 3-image | 19.01 (24.40) | 34.43 (85.50) | -3.00 | 0.003 |
| 5-image | 28.34 (45.45) | 33.84 (36.88) | -1.62 | 0.105 |
| 6-image | 30.16 (41.19) | 44.64 (68.41) | -3.14 | 0.002 |

Appendix Table 3: Completion Times with the Independent-elicitation Interface

| | Ordinal | Numerical | | |
|---|---|---|---|---|
| Task Size | Average (Standard Deviation) | Average (Standard Deviation) | $t$-statistic | $p$-value |
| 2-image | 18.66 (61.87) | 26.78 (74.84) | -1.45 | 0.149 |
| 3-image | 18.68 (25.86) | 33.96 (60.37) | -4.02 | <0.001 |
| 5-image | 27.46 (31.95) | 55.00 (76.36) | -5.75 | <0.001 |
| 6-image | 33.24 (41.36) | 72.23 (120.06) | -5.31 | <0.001 |
